# Military Surgeons in France

**Published:** 1850-10

**Authors:** 


### MILITARY SURGEONS IN FRANCE.—SPEECH OF M. DUPIN, PRESIDENT OF THE NATIONAL ASSEMBLY.

I have the honor of addressing the military surgeons of the French army, and I tell you, if any one were bold enough to still dispute your right to proportionate rank in the army, you might proudly answer by pointing to this statue, and by citing the life of the illustrious man whom it represents—the life of the worthy Larrey.

I have said it elsewhere, and I will repeat it whenever an opportunity offers—the military surgeon, fearless in epidemics, fearless in the field of battle, possesses all kinds of courage. He has military courage, because he faces death, offered on all sides by fire and sword; and another courage, far superior to this, for he preserves his calm coolness and presence of mind when his life is in the greatest peril. The blow which is aimed at him, and which he sees threatening, cannot, and even were he able, would not, be returned by him. He knows his hazardous situation, and does not hesitate to fulfil his dangerous duties.

Kneeling by the side of the disabled, with as firm a hand as when he is studying nature in the anatomical rooms, he dresses their wounds. But with these two kinds of courage he reaps two kinds of glory; and Larrey, who has shown his courage equally in both, now deserves to be honored with double glory. He has proved, when twice wounded, that the dangers which the military surgeon runs are not imaginary. He was wounded once in Egypt, in times of glorious memory, and another time at Waterloo, on that mournful day for France.

You heard, from those who addressed you before me, what the life of Larrey has been, and what services he has rendered to science. It is not for me to enter into the details of his noble career; I am, besides, not prepared for it; I speak, carried away by momentary impulse, and by the admirable speeches which have just been uttered. I judge this learned man—this defender of mankind—by considering, as a whole, his useful life, marked by the most enlightened and noble services, and I bow before this statue which so worthily represents him. Yes, I greet Larrey! the virtuous, devoted man; whose self-denial and devotion triumphed even over the elements, and who has been among us as an incarnation of genius and humane feelings. He deserves the thanks of science, the army, France, and the whole civilized world.

*Dublin Med. Press.*

